# The Optimal Starting Model to Search for the Accurate Growth Trajectory in Latent Growth Models

**DOI:** 10.3389/fpsyg.2018.00349

**Published:** 2018-03-27

**Authors:** Minjung Kim, Hsien-Yuan Hsu, Oi-man Kwok, Sunmi Seo

**Affiliations:** ^1^Quantitative Research, Evaluation, and Measurement, Department of Educational Studies, The Ohio State University, Columbus, OH, United States; ^2^Children's Learning Institute, University of Texas Health Science Center at Houston, Houston, TX, United States; ^3^Department of Educational Psychology, Texas A&M University, College Station, TX, United States; ^4^Department of Psychology, University of Alabama, Tuscaloosa, AL, United States

**Keywords:** latent growth models, latent curve models, growth analysis, model selection, specification search, starting model, model building, growth curve

## Abstract

This simulation study aims to propose an optimal starting model to search for the accurate growth trajectory in Latent Growth Models (LGM). We examine the performance of four different starting models in terms of the complexity of the mean and within-subject variance-covariance (V-CV) structures when there are time-invariant covariates embedded in the population models. Results showed that the model search starting with the fully saturated model (i.e., the most complex mean and within-subject V-CV model) recovers best for the true growth trajectory in simulations. Specifically, the fully saturated starting model with using ΔBIC and ΔAIC performed best (over 95%) and recommended for researchers. An illustration of the proposed method is given using the empirical secondary dataset. Implications of the findings and limitations are discussed.

## Introduction

Longitudinal data has been widely used in many research areas including medical, education, and psychology. One of the major questions when using longitudinal data is often on the change of the measured variables over time, such as: *are parental control and knowledge for their children declining over time?* (Keijsers and Poulin, 2013); *what are the developmental trajectories for adolescents' empathic concern associated with pubertal status?* (Van der Graaff et al., 2014). Most educational and psychological researchers are interested in not only the accurate growth, but also the factors/covariates (e.g., gender, involvement in peer-oriented leisure activities) accounting for the variation of growth trajectory among participants (Crockett and Beal, 2012; Titzmann et al., 2014). Latent growth models (LGM; also called latent growth curve models) have been increasingly popular in longitudinal studies given that the latent growth models allow researchers to take into account the between-individual differences as well as within-individual differences over time (Meredith and Tisak, 1990; Preacher et al., 2008; Duncan et al., 2013).

In longitudinal data analysis under LGM, many studies have devoted to optimally model the overall shape of the growth trajectories for all subjects based on the hypothesized model (Duncan et al., 1994; Hancock and Lawrence, 2006; Blozis, 2007). When there is no hypothesized theory, however, researchers may use exploratory approach to search for the optimal growth shape based on their data. Visual inspection using graphical function in statistical software (e.g., empirical growth plot) can be one viable approach to start with, but it is more suitable with a subset of sample rather than with a large sample data (Singer and Willett, 2003). While the traditional model building approach has been employed for decades, under this circumstance, there have been extensive efforts to suggest the model specification search strategy for the optimal shape of growth trajectory (Leite and Stapleton, 2011; Liu et al., 2012; Kim et al., 2016; Whittaker and Khojasteh, 2017). Under the framework of LGM, model specification search can be conducted in terms of the mean structure (i.e., shape of the overall changing pattern) and variance-covariance (V-CV) structure consisting of growth factor V-CV (i.e., variations across the individual growth trajectories) and residual V-CV structure (i.e., variations within the individual growth trajectories). Previous research has consistently found that the saturated residual variance-covariance structure (i.e., freely estimating the variance and covariances of repeated measures) has promising performance when searching for the accurate growth shape in simulations (Wu and West, 2010; Kim et al., 2016). However, existing recommendation has been made upon previous simulations, assuming that all growth latent factors are exogenous variables in the population models. That is, no studies have investigated whether existing recommendation is still applicable to the case that growth latent factors are both exogenous and endogenous variables at the same time. When the possible covariates are excluded in the model, the latent growth models is regarded to be *misspecified* given that the paths from the covariates to the growth factors are constrained to be 0. When the influential covariates are existing but not considered in the step of searching for the accurate growth trajectory, little is known about (a) which starting model performs best in searching for the optimal growth trajectory and (b) which model selection criteria can be used to successfully search for the best growth trajectory.

In the present study, we aim to investigate the optimal model search strategy for finding for the accurate growth shape in simulations when there is a significant covariate associated with the growth trajectory. Specifically, we focus on time-invariant covariates (e.g., gender, years of education, ethnicity) in the current study. Under the framework of LGM, we employ the four different starting models in terms of the complexity of mean and residual variance structure following the previous research by Kim et al. (2016): (1) the simplest mean and the error variance structure, (2) the most complex mean and the simplest residual variance structure, (3) the simplest mean and the most complex error variance structure, and (4) the most complex mean and the error structure. Specifically, we examine (1) which starting model performs best in model specification search, (2) which model evaluation index shows successful performance in finding the population growth shape, and propose the optimal model search strategy given the results of the two research questions. We use a Monte Carlo simulation study to investigate the effectiveness of different starting models on the search for the correct mean trajectory. An illustrative example is also presented to apply the model search strategy.

### Mean and residual variance (variance-covariance) structures in LGM

There are three model components in LGM: mean structure, between-subject variance-covariance (V-CV) structure, and within-subject V-CV structure. A general model formulation in LGM can be written as:

(1) $$\begin{array}{r} \text{y}=\tau_{\text{y}}+\Lambda_{\text{y}}\eta +\varepsilon , \end{array}$$

where y refers to a vector of outcome variables (t × 1, where t is the number of repeated measures), τ refers to a vector of intercepts of ys (t × 1; typically fixed to zero for model identification purpose), Λ represents a factor loading matrix for ys (t × *p*, where *p* is the number of latent growth factors), η is a vector of latent growth factors (*p* × 1), and ε represents a vector of errors for each y across the repeated measures (t × 1). η can be further written as follows:

(2) $$\begin{array}{r} \eta =\alpha +\Gamma_{\text{η}}\text{w}+\zeta , \end{array}$$

where α contains the vector of population initial status and growth parameters (e.g., $\left[\begin{array}{c} \alpha_{0}\\ \alpha_{1} \end{array}\right]$ for a linear growth model), Γ represents a matrix of regression coefficients of time-invariant covariate *w*, and ζ represents the deviation of the corresponding individual values from the mean estimates of those growth factors, respectively. Mean structure is the expected value of y [i.e., *E(y)* = Λ_y_α + Λ_y_Γ_η_E(*w*)^1^], which represents the average growth trajectory and the covariate effect on the change rates. In the current study, we aim to correctly search for the structure of growth shape (i.e., Λ_y_α) while omitting the covariate effect part [i.e., Λ_y_Γ_η_E(*w*)] by using an unconditional model in the search procedure. The variance-covariance (V-CV) of y in Equation (1) can be written as:

(3) $$\begin{array}{r} V\left(y\right)=\Sigma =\Lambda_{\text{y}}\text{Ψ}\Lambda_{\text{y}}^{\prime }+\Theta_{\text{ε}}, \end{array}$$

where Ψ is a p × p matrix containing the variance and covariances of the growth related latent factors; Λ'_y_ is the transpose of the Λ matrix which captures the overall pattern of change, and Θ_ε_ represents the matrix of variances and covariance among the errors (or unique factors). In other words, between-subject V-CV is captured by Ψ matrix, representing the differences on the intercepts and growth shapes among the subjects. In the current study, we focus on the specification of within-subject V-CV structure given that more complex structure and assumptions are associated with the within-subject V-CV components compared to between-subject V-CV structure (Kim et al., 2016). Within-subject V-CV structure (also called residual variance structure throughout this paper) is the variance and covariances of the repeated measures for each individual (t × t matrix of Θ_ε_), which captures the deviations of the observed variables from a vector of expected ys.

### Model building process in methodological studies

There have been a number of debates on how to search for the optimal growth shape in longitudinal data analysis. When there is no hypothesized growth shape in the absence of theory, there are two commonly used starting points in terms of the mean structure: the simplest mean structure (i.e., intercept-only model) and the most complex mean structure (i.e., highest possible polynomial growth model). The simplest intercept-only model has been frequently used for model search process in the longitudinal data analysis given that they follow the classic model search strategy provided by Raudenbush and Bryk (2002) from their classical book of hierarchical linear modeling.

Likewise, under the framework of multilevel model, Singer and Willett (2003) suggested starting from the unconditional model where there is a time-associated factor (e.g., age, year, months) but no other factors or covariates in the model. Similarly, McCoach and Kaniskan (2010) have demonstrated a model building method by starting with an unconditional linear growth model followed by adding time-varying covariates. In their study, the empirical data from 277 elementary school students over four time points are used for the demonstration.

Ryoo (2011) has conducted a simulation study for a model building approach and recommended of using the simplest mean structure model with no covariates as the starting point (i.e., intercept-only model) to search for the true growth shape. In his simulation study, six covariates are included for data generation while those are not included at the first step of model selection process. Static predictors of growth trajectories are introduced into the model at the last step after selecting the proper growth (mean) structure. Results show that the step-up (i.e., starting from the simplest mean structure) approach performs well to search for the true growth shape. Meanwhile, the error variance structure has not been discussed in the study and the default structure (i.e., simplest Identity structure) has been used for all simulation conditions.

On the other hand, under the framework of LGM, Mayer et al. (2012) has illustrated a 3-step model building process using a quadratic growth model as an example to show how to define the latent growth components in longitudinal data analysis. According to Mayer et al. (2012), based on a measurement model formulated at Step 1, specifying the saturated (most complex) mean structure is recommended for a starting model at Step 2 while covariates predicting growth components are added at Step 3.

In most studies, however, model specification for the variance-covariance structure part has been often disregarded in the model building process because it seldom impacts the shape of the growth trajectory itself (Kwok et al., 2007). However, the impact of ignoring the error variance structure gets more severe when conducting a model search because it may end up selecting an inaccurate growth shape as the best fitting model. Recently published study by Kim et al. (2016) shows that specifying the simplest within-subject V-CV structure, which is the default error structure in many statistical software, is less likely to select the optimal growth shape as the best fitting model. In their simulations, the average recovery rate for finding the population growth shape is <50% when using the simplest error variance structure, while it is above 85% when saturating the residual V-CV structure with using certain model evaluation criteria (e.g., LRT, ΔAIC, and ΔBIC). In their study, only unconditional models without covariates are used as a population model. There are no studies, at our knowledge, investigating the model specification search for the population growth shape in LGM, considering both mean and error variance structure when covariates are regressed on the growth factors.

### Applied studies using LGM

Many applied studies employing latent growth models under the multilevel modeling framework typically use the simplest within-subject V-CV structure (i.e., Identity [ID]; constant variance across repeated measures without allowing any covariance between the measures) because it is the default error variance structure in MLM software (e.g., SPSS MIXED, SAS PROC MIXED, HLM). Although there are published tutorials available for how to change the default within-subject error variance structure (or level-1 residual structure in MLM framework) (Quené and Van den Bergh, 2004), modifying the residual structure has been rarely considered in most applied research.

We reviewed substantive studies published in *Developmental Psychology* between 2010 and 2016 and found 37 studies^2^ employing the latent growth (or growth curve) models for the longitudinal data analysis. Among 37 studies, 15 studies specified a linear growth model with no search procedure due to the limited number of repeated measures (i.e., 3 waves). Among 22 of 37 studies containing 4 or more waves of data, 14 studies (63.6%) conducted a model comparison to find the best fitting growth trajectory while 8 studies directly specified their hypothesized growth shape (i.e., linear growth model for 7 studies and piece-wise growth model for one study). Among 14 studies conducting a model comparison, 8 studies contained 4 waves of data and they compared a linear growth model to a non-linear growth model (e.g., quadratic growth model). Among the rest of 6 studies, which conducted a model specification search with more than 4 waves of data, three studies reported the fit statistics (e.g., chi-square difference test, CFI, RMSEA, and SRMR) for all compared models. Nevertheless, none of studies reported the information regarding the specification of residual variance structure during the model search procedure. For the selected final model, majority of the studies (86.5%) directly specified the simplest residual variance structure without considering other types of V-CV structures. As shown in the reviewed literatures, there is a lack of consensus for using a model building approach in latent growth models to search for the optimal growth trajectory.

## Study aims

**Figure 1:**
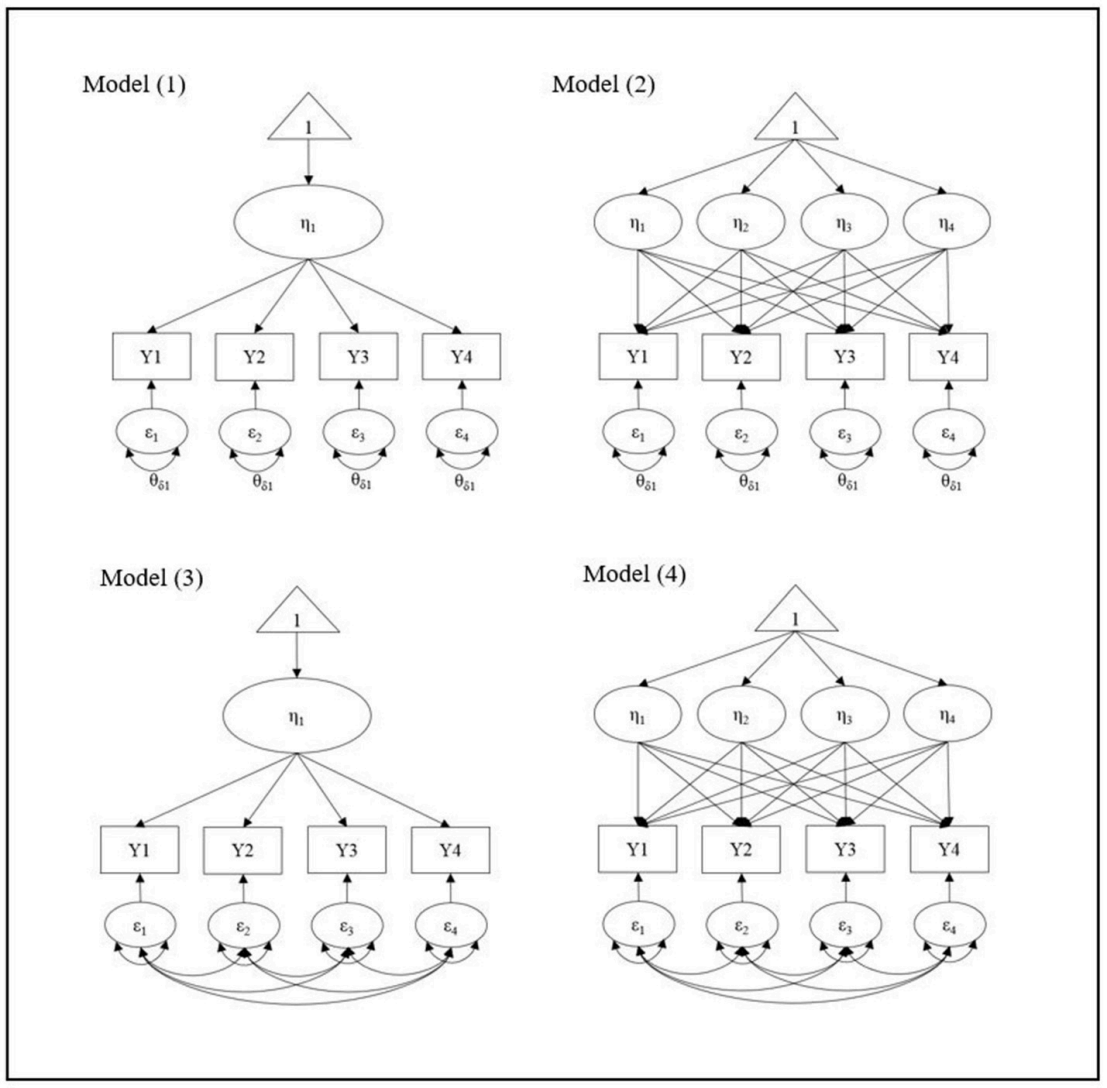
Four starting models for 4 wave data.

Our goal is to propose a universal starting model to search for the best-representing growth shape for the data regardless of the *true* population mean structure because, in reality, we do not know the true or accurate growth trajectory. We followed Kim et al. (2016) to set up the four starting models in terms of the mean and the residual structures in LGM. Figure 1 presents the four possible starting models for 4 wave data as an example: (1) the simplest mean (intercept-only) with the simplest ID error variance structure, (2) the most complex mean (e.g., highest possible polynomial growth term) and the simplest ID structure, (3) the simplest mean and the most complex UN error variance structure, and (4) the most complex mean and the error structure. We extend the previous study to consider more general conditions, in which there is a covariate effect on the growth trajectories. While it has been found that saturating the within-subject V-CV structure performs successfully to search for the true growth trajectory without considering covariates (Wu and West, 2010; Kim et al., 2016), the starting point for the mean structure has shown no consistent results. Given that the previous research used no covariates for the true model setting, we expand it to more general model with covariates and examine whether the consistent results can be found in more general conditions. We have two specific research questions in the current study.

Q1: Which starting model performs best in searching for the correct growth trajectory?

We examine the performance of four unconditional growth starting models to search for a population growth shape under the LGM framework. Based on the previous research, we hypothesize that the model specified with the most complex residual variance structure will perform successfully in searching for the growth shape. Given that the starting point for the mean structure has shown inconsistent results, we specifically interested in: *Does specification in mean structure (the most complex vs. the simplest) affect the recovery rate for detecting the true growth trajectory?*

Q2: Which model selection criteria performs successfully to search for the true growth trajectory?

We use six commonly used model evaluation criteria (i.e., LRT, ΔCFI, ΔRMSEA, ΔSRMR, ΔAIC, and ΔBIC) with two different model building approaches (i.e., step-up and top-down). We expect that LRT and two information criteria (i.e., ΔAIC and ΔBIC) will outperform the other fit indices based on the previous research finding (Kim et al., 2016).

## Methods: simulation study

### Data generation

**Figure 2:**
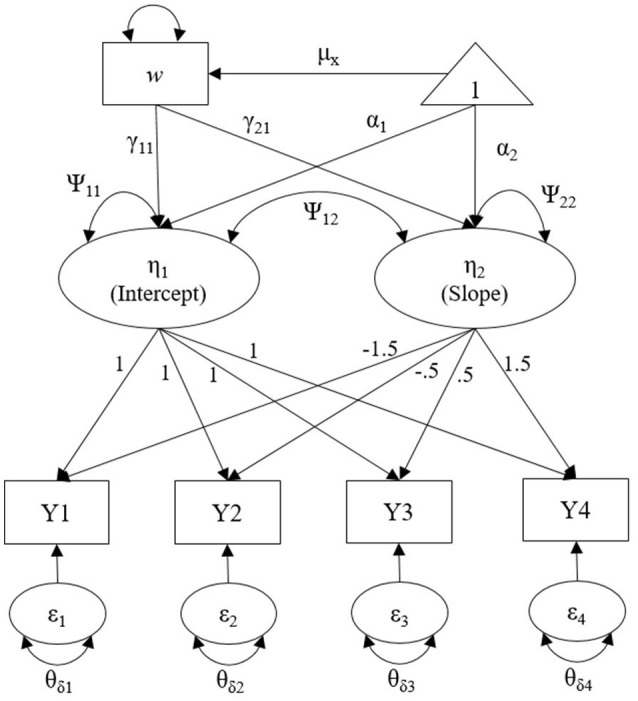
Linear growth model with UN(1) error variance structure with 4 waves of data to generate the true population model with a single covariate.

Data are generated using Mplus7.1 (Muthén and Muthén, 1998-2012) with a multivariate normal distribution. We have four major design factors in this simulation study: (a) 2 number of waves and mean structure (4 and 8 for linear and quadratic model, respectively), (b) true residual variance structure [ID, UN(1), and AR(1)]^3^, (c) 3 covariate effect sizes (0.1, 0.3, and 0.5), and (d) 3 sample sizes (100, 210, and 390), yielding a total of 54,000 datasets (2 × 3 × 3 × 3 × 1,000 replications). A thousand replications per simulation condition is reasonable for a simulation study in SEM given that many previous research have used equal to or fewer than 1,000 replications. Figure 2 shows an example of population model with 4 waves of data, which is a linear growth model with the UN(1) error variance structure. More details in simulation conditions for each design factor are described in the following section.

#### Number of waves and mean structure

We have used two conditions for the number of repeated measures, 4 and 8, for building up the population model of the growth trajectory. The number is based on reviewing the substantive studies published in developmental psychology between 2010 and 2016 as well as the previous simulation study (Kwok et al., 2007; Kim et al., 2016). The average number of waves used in longitudinal data analysis is 4.4 with a standard deviation of 1.6. Among a total of 37 reviewed studies employing the latent growth models, 24 studies have modeled a linear trend to analyze their data while 9 studies have used a quadratic growth trajectory to best represent their data. The rest of 4 studies have modeled their data other than linear and quadratic (e.g., piecewise growth model, factor loading freed non-linear model). Therefore, we have set up a population model of 4 waves of data to be a linear growth and 8 waves of data (i.e., approximately 2 standard deviations above the mean number of waves) to be a quadratic growth. Population values for both growth trajectories are set up to be a medium effect, which have been employed in the previous simulation studies (Kwok et al., 2007; Kim et al., 2016).

#### Residual variance structure

For generating the datasets representing the population model, we have used three types of variance-covariance structures, Identity (ID), Autoregressive [AR(1)], and banded main diagonal [UN(1)]^4^, which are commonly used in many longitudinal studies as well as simulation studies (Kwok et al., 2007; Kim et al., 2016). Among the 37 reviewed studies, 32 studies (86.5%) have used the simplest error V-CV structure (i.e., ID). Two studies have allowed a correlation between the error terms and 3 studies have estimated the time-specific variances [UN(1)]. The residual variances of the measurement waves (i.e., θ_δ_) were all set to be 1.00 for both ID and AR(1) structures, which was a common practice in power analysis and simulation studies. Following the prior simulation studies on residual variance structure, the autocorrelation coefficient, ρ, was set to be 0.50 for AR(1) structure. For the UN(1) structure, all the covariances were set to zero while the residual variance of the first time point was set to 1.00 and the following residual variances were set to be the power function of ρ = 0.80 (i.e., $\sigma_{1}^{2}$ = 1.00, $\sigma_{2}^{2}$ = 0.80, $\sigma_{3}^{2}$ = 0.64, and $\sigma_{4}^{2}$ = 0.51 for 4 waves of data; $\sigma_{1}^{2}$ = 1.00, $\sigma_{2}^{2}$ = 0.80, $\sigma_{3}^{2}$ = 0.64, $\sigma_{4}^{2}$ = 0.51, $\sigma_{5}^{2}$ = 0.41, $\sigma_{6}^{2}$ = 0.33, $\sigma_{7}^{2}$ = 0.26, and $\sigma_{8}^{2}$ = 0.21 for 8 waves of data), assuming that the reliability of the measurement increases over time (Grimm and Widaman, 2010).

#### Between-subject V-CV structure

We adopted the population parameters for the between-subject V-CV structure from the previous simulation studies in LGM (Kwok et al., 2007; Kim et al., 2016) Given that intercept variance has generally been larger than the variation of the change in growth in longitudinal studies (Raudenbush and Xiao-Feng, 2001), the total variance of Ψ_11_ was set to 0.20 while both Ψ_22_ and Ψ_33_ were set to 0.10 constantly for all conditions. The elements in the matrix were set to:

$$\begin{array}{l} \Psi_{\text{Linear}}=\left[\begin{array}{cc} \Psi_{11} & \Psi_{12}\\ \Psi_{21} & \Psi_{22} \end{array}\right]=\left[\begin{array}{cc} 0.20 & 0.05\\ 0.05 & 0.10 \end{array}\right] \end{array}$$

and

$$\begin{array}{l} \Psi_{\text{Quadratic}}=\left[\begin{array}{ccc} \Psi_{11} & \Psi_{12} & \Psi_{13}\\ \Psi_{21} & \Psi_{22} & \Psi_{23}\\ \Psi_{31} & \Psi_{32} & \Psi_{33} \end{array}\right]=\left[\begin{array}{ccc} 0.20 & 0.05 & 0.05\\ 0.05 & 0.10 & 0.035\\ 0.05 & 0.035 & 0.10 \end{array}\right] \end{array},$$

with the correlations (i.e., $r\;=\;\frac{\Psi_{xy}}{\sqrt{\Psi_{xx}\Psi_{yy}}},\;x\neq y$) setting as 0.35 for all the pairs of the elements in the Ψ matrix (Kwok et al., 2007). Based on the covariate effect sizes, the size of the variance and covariance of the growth associated factors was adjusted to consider the explained variance given the covariate.

#### Covariate effect size

To ease the understanding of mechanism under the model formulation with covariates, we have used a simple model with a single covariate in this simulation. Because adding more predictors is a function of increasing the total effect size, which decreases the size of residual (unexplained) variance, we have used three different sizes of covariate effects on the mean growth structure: 0.1 (small), 0.3 (medium), and 0.5 (large) while keeping a single covariate. The covariate, *w*, is generated to have a mean of 0 and a standard deviation of 1 with a normally distributed variance. The covariate effects are equally regressed on each growth-related term. For example, for the true linear growth model condition, a covariate has the same coefficient on the intercept and the slope.

#### Sample size

We have used three sample sizes, which are 100, 210, and 390, for small, medium, and large sample size conditions, respectively, following the previous simulation study (Kim et al., 2016). Thus, the total number of observations ranged from 400 (4 wave × 100 subjects) to 3,120 (8 wave × 390 subjects). We expect that the model stability will increase as sample size increases, indicating better chance of finding the correct mean trajectory.

### Evaluation criteria

We have evaluated three types of model selection criteria on the performance of finding the correct mean growth trajectory: (a) Likelihood Ratio Test (LRT), (b) ΔGoodness of Fit Indices [ΔGFI; i.e., Comparative Fit Index (CFI), Root Mean Square Error of Approximation (RMSEA), and Standardized Root Mean Residual (SRMR)], and (b) ΔInformation Criteria [ΔIC; i.e., Akaike Information Criteria (AIC) and Bayesian Information Criteria (BIC)]. These model selection criteria are commonly used by applied researchers because most statistical software for SEM provide these fit statistics in the output (e.g., Mplus, Lisrel, Amos). The difference in GFI and IC between the two competing models (i.e., constrained model vs. relaxed model) is calculated for each of the model fit index. Previous research has shown promising results for using both LRT and ΔIC in model specification search (Kim et al., 2016), whereas the ΔGFI showed inconsistent results. Following Kim et al. (2016), we have used the six model fit indices for evaluation criteria.

For information criteria (i.e., AIC and BIC), we have set up an absolute value to select a better fitting model over the competing model. Burnham and Anderson (2004) suggested using 4 for AIC to decide whether a model fit is significantly improved by adding additional parameter. Similary, Raftery (1995) suggested using 2 for BIC to compare the models. In other words, when the difference on the information criteria between the two competing models is minimal (i.e., ΔAIC < 4; ΔBIC < 2), we have selected the simpler model even when the more complex model showed smaller value of AIC and BIC. Similarly, we have adopted more stringent cutoff criteria for the GFIs proposed by Chen (2007). When the difference on CFI between the simpler and more complex model is < 0.01, the simpler model was selected over the more complex model. The cutoff for ΔRMSEA and ΔSRMR are 0.015 and 0.01, respectively.

### Model search process

For each dataset, four sets of model search procedure have been conducted using the four different starting models. Step-up refers to starting from the simplest mean structure (i.e., intercept-only model) by adding one more growth related factors at a time. For example, the intercept-only model with ID structure is compared to the linear growth model with the same residual variance structure using each of six different model evaluation criteria. If the model is significantly improved by adding a linear growth term, then the linear growth model is compared to the quadratic growth model in the next step, and so on. When the model is not improved any more, model search process is stopped and the simpler model between the two competing models is selected to be the optimal growth trajectory. If the selected growth trajectory is matched with the true (generated) growth structure, “hit” is coded as 1, while the incorrect growth trajectory is coded as 0. Since this process is independently conducted by six model evaluation criteria, the hit rates are varied across the model evaluation criteria. In a similar manner, top-down refers to starting from the most complex mean structure (i.e., cubic growth model for 4 wave; sextic (6th-order polynomial) growth model for 8 wave) by removing the highest growth related factor at a time. If the more complex model significantly fits better to the data, search has been stopped and the more complex model has been selected as the best fitting model.

### Dependent variable

The primary dependent variable was the hit rate of the true mean model being successfully identified by the model selection indices across the different starting models. For this dependent variable, correct model recovery was coded as a binary variable (i.e., 0 for a miss and 1 for a hit) for all replicates by all conditions. The hit rate (i.e., percentage of replicates reaching the true mean model) was summarized according to the performance of different starting models and model selection fit indices.

## Results: simulation study

Before using the model search process, we first analyzed the correctly specified model in terms of both mean and within-subject V-CV structures to validate the data generation process. Results show that all simulations for linear and quadratic growth models with the corresponding true error variance structures [i.e., ID, AR(1), and UN(1)] are properly converged with the accurate parameter estimates indicating that the data were adequately generated. Next, for each true model, four different starting models have been utilized to search for the true mean structure: (1) the simplest mean (intercept-only) with the simplest ID error variance structure, (2) the most complex mean (e.g., highest possible polynomial growth term) and the simplest ID structure, (3) the simplest mean and the most complex UN error variance structure, and (4) the most complex mean and the error variance structure. We present the results of our simulation studies by two research questions.

### Which starting model performs best in searching for the correct growth trajectory?

Table 1 presents the average hit rates (i.e., percentage of replicates reaching the true growth shape) across all six model fit evaluation criteria when using the four different starting models. Although each fit index is used independently for model search, we average the hit rates of all six fit indices to clearly compare the performance of the four starting models corresponding to the research question. The first three columns provide the information regarding the analyzed model using different starting points^5^. For the within-subject V-CV structure, ID [identity] is the simplest structure while UN [unstructured] is the most complex structure. For the mean structure in the next column, step-up refers to starting with the simplest mean (i.e., intercept-only model) while top-down refers to starting with the most complex mean model (i.e., cubic growth model for 4 wave data; 6th-order polynomial growth model^6^ for 8 wave data). The next two columns give the information about the true model conditions for the covariate effect size and sample size. The 6th to 11th columns report the average hit rates under six different true model conditions.

**Table 1:** Average percentage of finding the correct mean structure by four starting models.

						**Linear growth**			**Quadratic growth**		
**Starting model^a^**	**Cov spec**	**Mean spec**	**Effect size**	***n***	**Average hit**	**ID**	**UN(1)**	**AR(1)**	**ID**	**UN(1)**	**AR(1)**
(1)	ID	Step-up	0.1	100	56.0	76.1	65.6	51.1	64.0	49.8	29.3
(1)	ID	Step-up	0.1	210	48.1	80.9	57.5	31.7	65.1	36.7	16.8
(1)	ID	Step-up	0.1	390	41.2	83.9	45.5	14.2	65.3	25.6	12.6
(1)	ID	Step-up	0.3	100	57.7	78.3	68.3	51.5	64.4	53.8	29.9
(1)	ID	Step-up	0.3	210	50.2	81.6	61.6	32.2	65.3	42.1	18.1
(1)	ID	Step-up	0.3	390	43.3	84.5	50.7	14.4	65.1	30.9	14.2
(1)	ID	Step-up	0.5	100	58.6	79.4	70.1	51.9	64.9	53.5	31.8
(1)	ID	Step-up	0.5	210	51.1	82.6	65.1	32.8	65.4	40.9	20.0
(1)	ID	Step-up	0.5	390	44.6	86.1	56.2	14.6	65.1	29.3	16.0
Model (1) Average hit					50.1	81.5	60.1	32.7	64.9	40.3	21.0
(2)	ID	Top-down	0.1	100	56.6	71.3	62.2	49.3	86.3	41.8	28.7
(2)	ID	Top-down	0.1	210	50.7	76.5	55.6	31.0	91.7	32.5	16.7
(2)	ID	Top-down	0.1	390	45.5	80.0	44.9	14.1	93.9	27.7	12.2
(2)	ID	Top-down	0.3	100	58.5	73.6	64.8	49.6	86.7	45.7	30.5
(2)	ID	Top-down	0.3	210	52.7	77.2	59.7	31.4	91.9	35.9	20.2
(2)	ID	Top-down	0.3	390	47.6	80.9	50.1	14.3	94.1	30.4	15.8
(2)	ID	Top-down	0.5	100	60.4	74.7	66.6	50.1	87.5	49.1	34.6
(2)	ID	Top-down	0.5	210	55.2	78.5	63.2	31.9	92.1	40.1	25.6
(2)	ID	Top-down	0.5	390	50.7	83.0	55.5	14.4	94.0	35.9	21.6
Model (2) Average hit					53.1	77.3	58.1	31.8	90.9	37.7	22.9
(3)	UN	Step-up	0.1	100	73.8	81.0	84.1	83.3	66.7	69.1	58.7
(3)	UN	Step-up	0.1	210	83.4	88.3	89.1	91.5	79.5	80.6	71.6
(3)	UN	Step-up	0.1	390	86.3	90.6	91.5	93.7	83.6	83.8	74.4
(3)	UN	Step-up	0.3	100	64.2	85.7	86.4	88.3	43.0	44.6	37.4
(3)	UN	Step-up	0.3	210	73.4	88.8	89.8	91.7	58.2	59.0	52.6
(3)	UN	Step-up	0.3	390	77.9	91.4	92.4	93.8	64.6	64.3	60.6
(3)	UN	Step-up	0.5	100	52.0	86.7	87.4	89.2	16.8	16.9	15.1
(3)	UN	Step-up	0.5	210	58.1	89.8	90.8	91.9	25.5	26.8	23.6
(3)	UN	Step-up	0.5	390	65.8	92.3	93.1	94.0	38.6	39.9	36.6
Model (3) Average hit					70.5	88.3	89.4	90.8	52.9	53.9	47.8
(4)	UN	Top-down	0.1	100	76.6	67.8	70.7	71.2	82.8	83.8	83.3
(4)	UN	Top-down	0.1	210	82.5	75.0	75.9	80.8	87.2	88.1	87.7
(4)	UN	Top-down	0.1	390	85.3	78.7	79.8	84.9	89.4	89.3	89.7
(4)	UN	Top-down	0.3	100	78.8	72.4	72.9	76.2	83.2	84.2	83.8
(4)	UN	Top-down	0.3	210	82.9	75.7	76.8	81.1	87.5	88.2	87.9
(4)	UN	Top-down	0.3	390	85.8	79.8	81.0	85.2	89.4	89.4	89.7
(4)	UN	Top-down	0.5	100	79.5	73.2	73.8	77.0	83.7	84.6	84.4
(4)	UN	Top-down	0.5	210	83.5	76.9	77.9	81.6	87.8	88.5	88.1
(4)	UN	Top-down	0.5	390	86.2	81.0	81.9	85.7	89.5	89.5	89.7
Model (4) Average hit					82.3	75.6	76.7	80.4	86.7	87.3	87.1

a *Model (1): intercept-only with the simplest Identity V-CV structure, Model (2): highest-order polynomial growth (i.e., cubic for linear growth and sextic for quadratic growth population model) with the Identify V-CV, Model (3): intercept-only with the most complex UN V-CV structure, Model (4): highest-order polynomial growth with the UN V-CV structure*.

As shown in Table 1, starting model (4), which is specified with the most complex mean and residual variance structure, performs best in searching for the population growth trajectory. The average hit rate is 82.3% across all simulation conditions and all model selection criteria. The average hit rates for six different population models range between 75.6 and 87.3% indicating relatively stable performance across all simulation conditions. As covariate effect size and sample size increase, the percentage of finding the true growth shape slightly increases when using the starting model (4). Following Model (4), Model (3) that uses the intercept-only with the most complex UN structure as the starting point shows 70.5% of average hit rate with a range between 47.8 and 90.8%. While Model (3) performs relatively well for searching for the linear growth model (ranged between 88.3 and 90.8%), hit rates are substantially decreased for the quadratic growth model (ranged between 47.8 and 53.9%). As shown in Table 1, as covariate effect size increases, hit rates for Model (3) decreases across different sample size conditions and error variance structures. Results for each fit index show that the fit statistic difference between the intercept-only model and linear growth model is minimal, which leads to select the intercept-only model as the better fitting model than the linear growth model. Although Model (3) outperforms Model (4) for the true linear growth model, it shows unstable results for the true quadratic mean structure, which indicates that Model (3) is sensitive to the true mean structure while Model (4) is relatively robust to the true growth shape. More specifically, hit rates for Model (3) substantially decreases when the sample size becomes smaller and covariate effect size gets larger.

Meanwhile, Model (1), which is the most commonly used starting model in practice, shows the worst performance in searching for the accurate growth shape with overall average hit rate of 50.1% (ranged between 21.0 and 81.5%). Only when the true model is a linear growth model with the ID structure, Model (1) shows a good performance (81.5% hit rate). Given that not only the true mean structure is adjacent to the starting mean structure (i.e., a linear growth model and an intercept-only model) but also the V-CV structure is correctly specified (i.e., ID), it can be well expected that Model (1) performs successfully under this specific condition. Similarly, Model (2) (i.e., the most complex mean with the simplest V-CV structure model) shows no promising results in searching for the true mean structure with the average hit rate of 53.1% (ranged between 22.9 and 90.9%). Model (2) shows a good performance only when the true V-C structure is the true ID structure; the average hit rates are 88.0 and 90.9% for the linear and quadratic growth model, respectively. However, when the true V-CV structure is not ID but UN(1) or AR(1), both Model (1) and (2) show poor performance in detecting the true shape of the growth. Notably, Model (1) and (2) perform worse as sample size increases, which can be an evidence of the unstable model results (Kim et al., 2016).

### Which model selection index performs well in searching for the accurate growth shape?

Table 2 presents the average hit rates for six model selection fit indices across all simulation conditions. As shown in the table, ΔBIC shows the highest average hit rate (84.4%) across all simulation conditions followed by ΔAIC (average hit rate of 73.3%). The average hit rate of LRT across all simulations is 67.5% followed by ΔSRMR (57.4%), ΔCFI (53.6%), and ΔRMSEA (48.2%). Specifically, ΔBIC and ΔAIC using the starting Model (4) show outstanding performance to search for the true growth shape with the average hit rate of 97.1% (ranged between 96.8 and 97.7%) and 95.2% (ranged between 93.9 and 96.7%), respectively. As shown in Table 2, using ΔBIC for Model (4) shows consistently good performance regardless of other design factors, which are, true mean and covariance structure, covariate effect size, and sample size. Although LRT and ΔGFI (i.e., ΔCFI, ΔRMSEA, and ΔSRMR) show no advantages over the information criteria, when starting with Model (4), hit rates for ΔCFI and LRT notably increase with an average hit rate of 93.8 and 85.5%, respectively. In summary, ΔBIC and ΔAIC perform optimally to search for the accurate growth shape when starting with the most complex mean structure with the saturated error variance structure.

**Table 2:** Average percentage of finding the correct mean structure by different model evaluation criteria.

			**Linear growth**	**Quadratic growth**				
**Selection criteria**	**Starting model^a^**	**Overall average (%)**	**ID**	**UN(1)**	**AR(1)**	**ID**	**UN(1)**	**AR(1)**
LRT	(1)	53.1	95.3	65.2	29.3	94.4	28.9	5.6
	(2)	45.8	95.3	65.1	29.3	80.3	2.8	2.3
	(3)	85.6	94.9	95.1	95.0	76.6	78.3	73.9
	(4)	85.5	90.2	90.4	90.3	80.8	80.9	80.6
LRT Average		67.5	93.9	78.9	61.0	83.0	47.7	40.6
ΔCFI	(1)	20.9	75.1	33.4	16.9	0.0	0.0	0.0
	(2)	43.4	66.0	28.4	15.9	98.5	29.0	22.5
	(3)	56.3	83.0	86.2	94.9	33.6	23.8	16.4
	(4)	93.8	83.0	86.2	94.9	99.5	99.7	99.8
ΔCFI Average		53.6	76.7	58.5	55.6	57.9	38.1	34.7
ΔRMSEA	(1)	31.1	84.3	72.2	29.7	0.2	0.0	0.0
	(2)	55.0	74.6	67.5	26.2	82.9	62.1	16.6
	(3)	49.5	83.9	83.9	83.8	15.0	17.9	12.5
	(4)	57.4	56.4	56.3	56.6	58.4	58.3	58.5
ΔRMSEA Average		48.2	74.8	70.0	49.1	39.1	34.6	21.9
ΔSRMR	(1)	54.7	35.3	18.6	8.5	96.4	91.5	77.9
	(2)	47.9	30.8	17.4	8.1	89.3	81.6	59.9
	(3)	63.9	71.2	72.9	74.5	53.4	59.5	52.0
	(4)	63.0	28.9	30.8	45.0	89.3	92.4	91.4
ΔSRMR Average		57.4	41.5	34.9	34.0	82.1	81.3	70.3
ΔAIC	(1)	61.0	98.1	77.7	40.9	98.5	40.8	10.1
	(2)	53.7	96.7	76.6	40.3	94.3	8.3	6.4
	(3)	83.3	97.4	98.1	97.4	69.2	71.5	66.0
	(4)	95.2	96.0	96.7	96.0	93.9	94.3	94.1
ΔAIC Average		73.3	97.1	87.3	68.6	89.0	53.7	44.1
ΔBIC	(1)	83.5	99.0	98.4	81.2	100.0	86.8	35.8
	(2)	75.6	98.3	97.6	80.7	98.5	45.7	33.1
	(3)	81.2	97.6	98.6	97.7	64.8	67.5	61.1
	(4)	97.1	96.9	97.9	97.0	96.8	97.0	97.0
ΔBIC Average		84.4	97.9	98.1	89.1	90.0	74.3	56.7

a *Model (1): intercept-only with the simplest Identity V-CV structure, Model (2): highest-order polynomial growth (i.e., cubic for linear growth and sextic for quadratic growth population model) with the Identify V-CV, Model (3): intercept-only with the most complex UN V-CV structure, Model (4): highest-order polynomial growth with the UN V-CV structure*.

## Applied study

To illustrate the use of the proposed model search strategy, we have examined the longitudinal trajectories of depressive symptoms among Mexican American elders in the U.S. using the Hispanic Established Population for Epidemiological Studies of the Elderly (EPESE), which is retrieved from Inter-university Consortium for Political and Social Research (ICPSR). The first wave of interviews was conducted between September 1993 and June 1994 (Markides, 1993-1994), with 3,050 Mexican Americans aged 65 and over residing in the five southwestern states that contain the majority of Mexican Americans: Texas, California, New Mexico, Colorado and Arizona. Follow-up interviews were then conducted approximately every 2–3 years, with a supplemental sample from the same cohorts as the original sample added in wave 5. Literature has shown that limited English proficiency (LEP) is frequently reported to be associated with more depression among immigrants because language barriers can be a significant source of stress (Nwadiora and McAdoo, 1996; Constantine et al., 2004; Sadule-Rios, 2012). Kim et al. (in press) have investigated whether LEP is a significant factor associated with the longitudinal trajectory of the depressive symptoms using a latent growth model. In the current demonstration, we illustrate the model search procedure using the EPESE data to search for the optimal growth shape of the depressive symptoms for older immigrants.

Specifically, we have used a total of six waves of data for the depressive symptoms, which are measured with the Center for Epidemiologic Studies Depression Scale (CES-D), a 20-item self-administered questionnaire (Radloff, 1977), in the EPESE between 1993 and 2007. Respondents were asked to assess the frequency of depressive symptoms experienced during the past week, based on a 4-point scale with categories in the subsequent order: rarely or none of the time (0), some or a little of the time (1), much of the time (2), and most or all of the time (3). The total scores for 20 items potentially ranged from 0 to 60, with higher scores indicating more depressive symptoms. Among a total of 3,952 participants, 602 respondents who have all six waves of data for CES-D are included in the further analysis. Mplus 7.3 using Maximum Likelihood estimation method (ESTIMATOR = MLR) was utilized for handling non-normality of the depression scores.

To search for the optimal growth trajectory, we analyzed a series of unconditional latent growth models (i.e., without having covariates) by changing the shape of the growth and compared the adjacent growth models using the information criteria (i.e., ΔBIC and ΔAIC), which showed the best hit rate for selecting the true population growth trajectory in simulations. Other fit indices (i.e., CFI, RMSEA, and SRMR) were also considered to meet the absolute fit criteria. First, based on our finding from the simulation study above, we specified the most complex (saturated) within-subject V-C structure (i.e., UN structure), which allows to freely estimate all the variance and covariance components. For the mean structure, we started with the quartic (i.e., 4th-order polynomial) growth model as the most complex mean structure with leaving one degree of freedom to generate the fit statistics for 6 waves of data^7^. Next, the quartic growth model with the UN error variance structure was compared with the cubic growth model, which has one less parameter in the mean structure to estimate. If there is no significant difference between the two competing models, we selected the simpler (cubic) growth model over the more complex (quartic) growth model. Next, the cubic growth model was compared to the quadratic growth model by eliminating the next highest-order polynomial growth term, and so on. When the model fit significantly got worse (i.e., ΔBIC > 2 and ΔAIC > 4), the model search was stopped and the more complex model was selected as the best fitting model.

**Figure 3:**
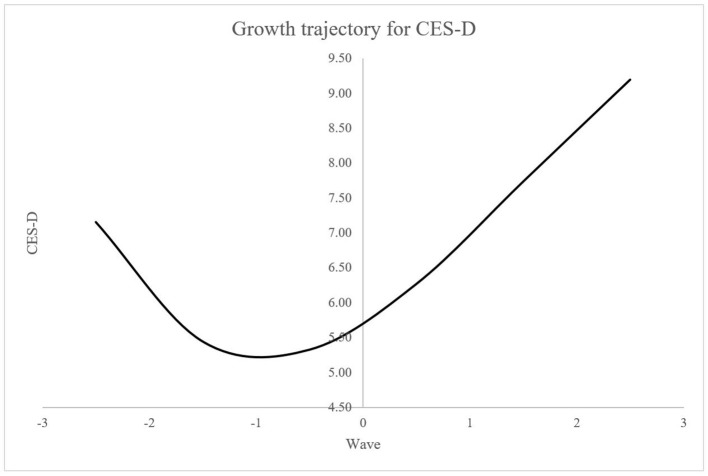
Cubic growth trajectory for depressive symptoms using CES-D measure for EPESE data.

Table 3 presents the model fit indices including the AIC and BIC for the series of latent growth models for the CES-D measures. As shown in Table 3, the cubic growth model was selected as the best fitting model by both information criteria. Using the ΔAIC, the quartic growth model shows no improvement from the cubic growth model, whereas the cubic growth model significantly better fits to the data than the quadratic growth model. Likewise, the cubic growth model is selected over the quartic growth model using the ΔBIC, and then, the cubic growth model is compared to the quadratic growth model, and indeed, the cubic growth model shows the better fit. Interestingly, the cubic growth model was selected by both step-up approach (i.e., starting from the intercept-only model) and top-down approach (i.e., starting from the quartic growth model) when specifying the saturated UN error variance structure in the current example. In other words, both starting points (i.e., simplest and the most complex) in terms of the mean structure reached to the same result in selecting the cubic growth model as the best fitting model. Results show that older immigrants' depressive symptoms have been decreased during the first two waves of data and then increased for the following four waves of data (see Figure 3). Further investigation and implication of the findings should be referred to the work by Kim et al. (in press).

**Table 3:** The AIC and BIC for unconditional growth models for EPESE data.

	**Quartic**	**Cubic**	**Quadratic**	**Linear**	**Intercept**
AIC	24,400	24,400	24,407	24,478	24,509
BIC	24,514	24,510	24,513	24,579	24,606

## Discussions

The purpose of the current study is to explore the optimal model search strategy for searching for the best-fitting growth trajectory in latent growth models (LGM). While starting with the unconditional model without covariates has been known to be a classical method for model building in longitudinal data analysis (Meredith and Tisak, 1990; Singer and Willett, 2003), there is a lack of research incorporating both mean and residual variance structure in model search process under the framework of LGM. In the current study, we expanded the previous simulation study by Kim et al. (2016) by considering the time-invariant covariates on the growth trajectory to provide a model search strategy under more general conditions. We specifically examined two research questions: (a) which starting model performs best in searching for the correct growth trajectory, and (b) which model selection index performs best in identifying the true growth shape. Based on the results of the simulation study, we found that (a) starting with the *fully saturated model* with the most complex mean structure as well as the most relaxed (unstructured) error variance structure, and (b) using the information criteria (i.e., ΔBIC and ΔAIC) over the other fit evaluation criteria (i.e., LRT and ΔCFI, ΔRMSEA, and ΔSRMR) performed best in search for the population growth shape in LGM.

To examine the first research question, we have compared the four starting models in terms of the complexity of the mean structure and the within-subject variance-covariance (V-C) structure in LGM (Figure 1). For the within-subject V-CV structure, results of the simulations have shown that starting with the most complex (saturated) structure (i.e., Model 3 and 4) best recovers the true growth shape across all simulation conditions, which is a consistent finding with the previous simulation study (Kim et al., 2016). Unlike the previous study, however, the current results show that starting point in the mean structure also does matter to successfully search for the true growth trajectory. When there is a small to moderate effect of covariates regressed on growth trajectories, starting with the most complex mean structure outperforms the simplest mean structure to recover the true growth shape. This new finding is important because many applied research have been using the simpler starting model (e.g., intercept-only model or linear growth model) to search for the possibly more complex growth trajectory (e.g., quadratic growth or cubic growth model) in practice. Based on the simulation results, if the simpler growth model is used as the starting point, they are more likely to select the incorrectly simpler growth model, which may not represent their data adequately. As shown in the results of the simulation study (Table 1), when the true growth trajectory is a quadratic growth, the average hit rate of the fully saturated model (Model 4) is 87.0% while the hit rate of the simplest mean with the most complex error variance structure model (Model 3) is 51.5%. As sample size gets smaller and the covariate effect size gets larger, the impact of the starting point in the mean structure on recovering the true model becomes more substantial.

On the other hand, when the simplest ID structure is specified for the within-subject V-CV, neither step-up method nor top-down method successfully recovers the population growth trajectory except when the population model is generated to have the ID structure. Specifically, we note that the most commonly used intercept-only model with the simplest within-subject V-CV structure performs poorly to search for the correct mean trajectory, which is a consistent finding with the previous simulation study (Kim et al., 2016). When the true within-subject V-CV is not Identity but more complex structure (i.e., UN(1) or AR(1) in the current study), the average hit rate even decreases as sample size increases, which is another evidence of model instability. Given that researchers do not know the population or true variance structure in reality, specifying the simplest V-CV structure with no further consideration should be avoided in the model search process based on the current research finding.

To examine the second research question, we have used six model fit indices, which are LRT, ΔCFI, ΔRMSEA, ΔSRMR, ΔAIC, and ΔBIC to select for the best fitting growth trajectory model. Results show that there is no single fit index performing consistently well across all starting models. On the other hand, ΔBIC and ΔAIC performed successfully to search for the accurate growth trajectory with the use of the most complex starting model. As shown in the Appendix, average hit rates of ΔBIC and ΔAIC with using the Model (4) are above 95% on average across all simulation conditions. That being said, when researchers search for the optimal growth trajectory in LGM, starting with the most (or possibly more) complex mean structure with relaxing any constraints on error V-CV structure is highly recommended.

## Limitations and future

The current study has several limitations in study designs and conditions as with most simulation studies. First, we limited our study conditions for polynomial one-piece growth models (e.g., linear and quadratic growth models) in simulations based on the literature review, where majority of the applied research employed the polynomial growth models. While starting with a polynomial growth model is a reasonable approach, the proposed method might perform differently when the best model is a family of exponential growth models or piecewise growth models. Since the existence of multiple-piece non-linear model (e.g., piecewise exponential growth) is possible in reality, further research on the effectiveness of current approach with more complex multiple-piece models is needed. In addition, when the number of repeated measures is 3, this approach may not be adequate due to the limited number of testable growth models (intercept only, linear and quadratic).

Next, we used a single covariate with effect sizes to be equally regressed on all time factors (e.g., intercept and linear for a linear growth model). Some predictors may have a stronger effect on the initial time measure (e.g., intercept) than on the changing rate (e.g., linear and quadratic growth factors) or vice versa. Moreover, when there are multiple covariates or factors, models get easily complicated with possible interaction effects and effect sizes differed. We simplified the simulation conditions to use the constant effect sizes for the single covariate so that we can examine the effect of time-invariant covariates in model search process more clearly.

In the current study, we only considered the predictor(s) to be time-invariant covariates (e.g., gender, age, years of education, etc.) by excluding the scenarios for the time-varying covariates. Moreover, we have limited the assumption for the time-invariant covariates to be fully mediated by the growth parameters at the subject level. That is, we have assumed that the direct effect of time-invariant covariate on each time measure is equal to zero, which is regarded as a more standard way to model the time-invariant covariates in LGM (Whittaker and Khojasteh, 2017). While we believe that the current findings can be applied to more complex situations, further research is warranted to investigate the generalizability of the current research finding to more general conditions including the time-varying covariates in the population model.

This study has focused on the model specification search for finding for the accurate growth trajectory while having the search process for the residual variance structure left questionable. Given that the misspecified error variance structure has detrimental impacts on the inferences about growth parameters (Ferron et al., 2002; Kwok et al., 2007), searching for the correct or adequate error variance structure should be followed by specifying the optimal growth trajectory. Recently published simulation study by Ding et al. (2017) has provided a systematic approach to facilitate identifying a plausible covariance structure. Although they have conducted a study based on unconditional growth models, the guideline given in the study can be used as another starting point for searching the adequate error variance structure in LGM.

### Implications and practical recommendations

Latent growth models are a popular method for longitudinal data analysis for decades given the flexibility of modeling the within- and between-subject error variance structure. This simulation study has investigated the performance of different starting models to search for the best-fitting growth trajectory in LGM under more general conditions than the previous simulation study. In the absence of certainty for the growth trajectory, the current study proposes to use the most complex (fully saturated) starting model with the highest-order polynomial growth factors and the most relaxed error variance structure, which performed the best to search for the true growth trajectory. Among the widely used fit indices for model comparisons (i.e., LRT, ΔGFI, and ΔIC), ΔBIC and ΔAIC with using the fully saturated starting model showed the most promising results in detecting the population growth trajectory over other fit indices. Based on the optimally specified growth trajectory, researchers should follow the next steps for model building process, such as, modeling the time-invariant and time-varying predictors, moderating effects, and specifying the proper covariance structures, to best understand the data and to examine their research questions.

## Author contributions

All authors listed have made a substantial, direct and intellectual contribution to the work, and approved it for publication.

### Conflict of interest statement

The authors declare that the research was conducted in the absence of any commercial or financial relationships that could be construed as a potential conflict of interest.

## Footnotes

^1^A vector of τ_y_ is fixed to zero for model identification purpose.

^2^A list of studies is provided on the first author's website as an Appendix.

^3^ID = Identity; UN(1) = Banded main diagonal; AR(1) = Autoregressive.

^4^ID $\text{ID}=\sigma^{2}\left[\begin{array}{cccc} 1 & \; & \; & \;\\ 0 & 1 & \; & \;\\ 0 & 0 & 1 & \;\\ 0 & 0 & 0 & 1 \end{array}\right];\text{AR}(1)=\sigma^{2}\left[\begin{array}{cccc} 1 & \; & \; & \;\\ \rho & 1 & \; & \;\\ \rho^{2} & \rho & 1 & \;\\ \rho^{3} & \rho^{2} & \rho & 1 \end{array}\right];\text{UN}(1)=\left[\begin{array}{cccc} \sigma_{1}^{2} & \; & \; & \;\\ 0 & \sigma_{2}^{2} & \; & \;\\ 0 & 0 & \sigma_{3}^{2} & \;\\ 0 & 0 & 0 & \sigma_{4}^{2} \end{array}\right]$.

^5^Given the limited space, we present the summary of the simulation results. Result tables for six true model conditions including covariate effect size and sample size information are available from the first author upon request.

^6^Instead of the seventh-order polynomial model, we used the sixth-order polynomial model as the most complex mean model given that the seventh-order polynomial model resulted in serious nonconvergence issue.

^7^Fully saturated model in both mean and V-CV structures is just-identified model and generates no fit evaluation statistics other than the information criteria.
