# Supplementary material for: The Optimal Starting Model to Search for the Accurate Growth Trajectory in Latent Growth Models
Source: Front Psychol. 2018 Mar 27;9:349. doi: 10.3389/fpsyg.2018.00349 (PMC5880923; doi:10.3389/fpsyg.2018.00349)
Supplement: Supplementary file 1 [file Table1.PDF]

Appendix. Hit rates of finding the true mean structure when using the starting Model (4) with ΔBIC and ΔAIC as model selection criteria

	Covariate effect size	Sample size	average	Linear growth ID	Linear growth UN(1)	Linear growth AR(1)	Quadratic growth ID	Quadratic growth UN(1)	Quadratic growth AR(1)
ΔBIC	0.1	100	91.5	85.5	92.5	85.9	94.9	95.2	95.1
	0.3	100	96.3	97.3	97.6	97.5	94.8	95.3	95.2
	0.5	100	96.6	98.0	98.0	98.2	94.8	95.1	95.3
	0.1	210	98.1	98.6	98.8	98.4	97.6	97.7	97.7
	0.3	210	98.1	98.7	98.7	98.7	97.5	97.7	97.7
	0.5	210	98.2	98.6	98.5	98.8	97.6	97.9	97.6
	0.1	390	98.7	99.2	99.2	99.0	98.3	98.2	98.5
	0.3	390	98.8	99.2	99.2	99.0	98.4	98.4	98.5
	0.5	390	98.8	99.2	99.2	99.0	98.5	98.3	98.5
ΔAIC	0.1	100	91.3	87.5	93.1	87.5	93.4	93.2	93.1
	0.3	100	95.1	96.6	97.2	96.8	93.3	93.4	93.2
	0.5	100	95.1	97.4	97.2	97.5	92.5	93.2	93.1
	0.1	210	93.8	93.8	95.8	93.9	93.0	93.2	93.1
	0.3	210	95.9	97.5	97.5	96.9	94.1	94.9	94.3
	0.5	210	95.8	97.4	97.4	97.0	94.2	94.9	94.3
	0.1	390	95.8	97.1	97.4	97.0	94.3	94.9	94.3
	0.3	390	95.8	97.3	97.4	97.0	94.2	94.9	94.3
	0.5	390	96.1	97.2	97.3	97.4	94.5	95.0	95.2
